# Indocyanine green (ICG) guided thyroidectomy versus conventional thyroidectomy and the impact on parathyroid function preservation: an updated GRADE-assessed systematic review and meta-analysis

**DOI:** 10.1007/s00423-026-04210-3

**Published:** 2026-09-01

**Authors:** Ahmed Wael Moftah, Abdo Mohamed Abdalhy, Hammam Majed Alsousi, Abdalrahman Salah Shehata, Mahmoud Ashraf Hussein, Yasser Ashraf Gamal, Myame Ayman, Israa A. Solah, Hebatalla Aly

**Affiliations:** 1 Faculty of Medicine, King Salman International University, South Sinai, Egypt; 2 Faculty of Medicine, Suez Canal University, Ismailia, Egypt; 3 Faculty of Medicine, University of Al-Butana, Gaza, Palestine; 4 Faculty of Medicine, South Valley University, Qena, Egypt; 5 Faculty of Medicine, Capital University, Cairo, Egypt; 6 Faculty of Medicine Assiut University, Asyut, Egypt; 7 Faculty of Medicine, Al-Yarmouk university, Irbid, Jordan; 8 Faculty of Medicine, Menoufia University, Menoufia, Egypt

**Keywords:** Thyroidectomy, Near-infrared autofluorescence, Indocyanine green, Parathyroid preservation, Hypoparathyroidism, Hypocalcemia

## Abstract

**Background:**

Thyroidectomy carries a significant risk of parathyroid gland injury, with transient hypoparathyroidism occurring in 15–50% of cases and permanent hypoparathyroidism in up to 5%, resulting in lifelong morbidity. Near-infrared (NIR) autofluorescence imaging combined with indocyanine green (ICG) fluorescence angiography has emerged as an intraoperative adjunct for parathyroid identification and perfusion assessment, but its comparative efficacy over conventional white-light surgery remains to be comprehensively quantified.

**Methods:**

A systematic search across five databases through January 2026 identified randomized controlled trials and observational studies comparing NIR autofluorescence with ICG imaging versus conventional thyroidectomy without fluorescence guidance. Outcomes included inadvertent parathyroid removal, permanent and transient hypoparathyroidism, permanent and transient hypocalcemia, postoperative parathyroid hormone (PTH) levels, postoperative serum calcium levels, and operative time. Meta-analysis utilized a random-effects model to calculate pooled risk ratios (RRs) and mean differences (MDs) with 95% confidence intervals (CIs). Certainty of evidence was assessed using the GRADE approach.

**Results:**

Eleven studies encompassing 2,203 patients (ICG: *n* = 897; Control: *n* = 1,306) were included. ICG-guided NIR imaging significantly reduced inadvertent parathyroid removal (RR 0.43, 95% CI 0.30–0.61, *p* = 0.002; I²=0%) and permanent hypocalcemia (RR 0.25, 95% CI 0.17–0.37, *p* = 0.004; I²=0%). A non-significant trend toward reduced permanent hypoparathyroidism was observed under our pre-specified random-effects model (RR 0.45, 95% CI 0.20–1.02, *p* = 0.054). A post-hoc sensitivity analysis using a fixed-effect model yielded a statistically significant result (RR 0.50, 95% CI 0.36–0.71, *p* < 0.001); however, this finding should be interpreted with caution, as it did not correspond to our primary pre-specified analytical approach. ICG guidance was associated with significantly higher postoperative PTH levels (MD 5.45 pg/mol, 95% CI 0.26–10.64, *p* = 0.042) and serum calcium levels (MD 0.06 mmol/L, 95% CI 0.01–0.11, *p* = 0.033). No significant differences were noted in transient hypoparathyroidism (RR 0.56, 95% CI 0.21–1.53, *p* = 0.185) or transient hypocalcemia (RR 0.67, 95% CI 0.33–1.34, *p* = 0.182), both characterized by substantial heterogeneity (I²=78.4% and 75.4%, respectively). Operative time was comparable between groups (MD 0.08 min, 95% CI − 18.25 to 18.42, *p* = 0.989).

**Conclusions:**

NIR autofluorescence with ICG imaging is associated with a reduction in inadvertent parathyroid removal and permanent hypocalcemia, supported by high and moderate certainty evidence respectively, along with improvements in postoperative PTH and calcium levels (moderate certainty). Evidence for benefit in permanent hypoparathyroidism was directionally favorable but of low certainty, and findings for transient hypoparathyroidism and transient hypocalcemia were inconclusive, rated low to very low certainty due to substantial heterogeneity. These findings support ICG-guided NIR imaging as a promising intraoperative adjunct, with the strongest evidence for reducing inadvertent gland removal and permanent hypocalcemia specifically, rather than uniform benefit across all parathyroid-related outcomes. Future large-scale randomized trials with standardized imaging protocols, blinded outcome assessment, and extended follow-up are needed to resolve heterogeneity in transient outcomes and confirm long-term benefits in high-risk surgical populations.

**Supplementary Information:**

The online version contains supplementary material available at https://doi.org/10.1007/s00423-026-04210-3.

## Introduction

Thyroidectomy is one of the most performed endocrine surgical procedures worldwide, serving as the standard approach for a broad spectrum of thyroid pathologies including malignancy, multinodular goiter, and refractory hyperthyroidism. However, it carries a risk of inadvertent parathyroid gland injury, devascularization, or excision, resulting in postoperative hypocalcemia and hypoparathyroidism ranging from 15% to 50% for transient cases and up to 5% for permanent hypoparathyroidism [1, 2]. Transient hypoparathyroidism causes neuromuscular irritability, prolonged hospitalization, and the need for calcium and vitamin D supplementation, while permanent hypoparathyroidism defined as an ongoing need for calcitriol supplementation to maintain normocalcemia at 12 months postoperatively, carries a substantial burden of lifelong supplementation, impaired quality of life, and complications including nephrocalcinosis, nephrolithiasis, renal insufficiency, cataracts, seizures, cardiac arrhythmias, ischemic heart disease, and depression [3, 4]. These complications arise primarily from inadvertent injury, devascularization, or excision of parathyroid glands that are notoriously difficult to identify intraoperatively due to their small size, variable anatomy, and resemblance to surrounding fibroadipose and lymphatic tissue. Conventional intraoperative identification relies predominantly on the operating surgeon’s visual recognition, an inherently subjective process susceptible to inter-operator variability and providing no functional information regarding gland perfusion [7, 8]. This leaves surgeons unable to predict which glands are at risk of postoperative dysfunction following dissection or ligation of feeding vessels, a limitation that is particularly consequential in complex dissections, reoperative fields, and extensive central compartment lymphadenectomy [9]. To address these limitations, near-infrared (NIR) fluorescence imaging technologies have emerged as intraoperative adjuncts for parathyroid identification and vascular assessment. NIR autofluorescence (NIRAF) exploits the intrinsic optical properties of parathyroid tissue to enable real-time, label-free identification, while indocyanine green (ICG) fluorescence angiography allows dynamic assessment of parathyroid perfusion, guiding intraoperative decisions regarding reimplantation or preservation [5, 6]. Together, this dual-modality approach offers both anatomical localization and functional perfusion assessment within a single operative workflow and has been associated with reduced postoperative hypocalcemia, permanent parathyroid dysfunction, and inadvertent gland resection compared with conventional visual identification [10, 11]. Therefore, this updated systematic review and meta-analysis was conducted to comprehensively evaluate the effectiveness of near-infrared autofluorescence combined with ICG fluorescence imaging compared with conventional thyroidectomy in improving parathyroid gland preservation and reducing postoperative hypoparathyroidism and hypocalcemia. By synthesizing evidence from both randomized controlled trials and observational studies and applying GRADE methodology, we aimed to clarify whether NIR autofluorescence with ICG imaging represents a clinically meaningful advance in reducing the morbidity associated with inadvertent parathyroid injury during thyroid surgery.

## Materials and methods

### Protocol and registration

The study methodology and results were reported following the Preferred Reporting Items for Systematic Reviews and Meta-Analyses (PRISMA 2020) statement guidelines [12]. The study protocol was registered on PROSPERO (*CRD420261348529).*

### Search strategy and study selection

A comprehensive literature search was conducted utilizing the core concepts of our research question across PubMed, Clinical trials.gov, Scopus, Web of Science and the Cochrane Library to identify studies published up to January 2026. Our search was restricted to English-language publications, given resource constraints for translation and quality assessment of non-English studies. No restriction was placed on publication status and both peer-reviewed published studies and gray literature were eligible for inclusion; ClinicalTrials.gov was searched specifically to identify unpublished and ongoing trials. In addition to the database search, we manually screened the reference lists of all included studies and relevant prior systematic reviews and meta-analyses to identify additional eligible studies not captured by the primary search strategy. Records identified through all sources were imported into Rayyan for de-duplication and screening. Duplicate records were identified and removed by manual review. Title and abstract screening, followed by full-text review, were performed independently by two reviewers, with disagreements resolved through discussion or adjudication by a third reviewer. The complete, line-by-line search strategy tailored for each database is detailed in the supplementary material The complete, line-by-line search strategy tailored for each database is detailed in the supplementary material (Supplementary Table S1).

### Eligibility criteria

We included published randomized controlled trials (RCTs) and observational studies on our topic. Included studies were eligible if they enrolled participants undergoing thyroid surgery with reported parathyroid-related outcomes. The intervention of interest was any form of near-infrared fluorescence imaging used intraoperatively to aid parathyroid identification and/or perfusion assessment. This encompassed three related but mechanistically distinct approaches: (1) near-infrared autofluorescence (NIRAF) alone, (2) indocyanine green (ICG) fluorescence angiography alone, and (3) combined dual-modality NIRAF + ICG imaging. Although these techniques differ in their primary mechanism (anatomical localization versus functional perfusion assessment), they share the common clinical objective of improving intraoperative parathyroid preservation and were therefore pooled under a single intervention category for the primary analysis. A detailed classification of each included study according to the specific fluorescence modality employed is provided in (Supplementary Table S4).

### Quality assessment of included studies

The Newcastle-Ottawa Scale (NOS) [13] was used to assess the quality of included observational studies. The NOS awards stars across three domains: Selection, Comparability, and Outcome. The total score ranges from 0 to 9, with higher scores indicating a lower risk of bias. The NOS scores were categorized into Agency for Healthcare Research and Quality (AHRQ) categories: Good (7 to 9 stars), Fair (5 to 6 stars), and Poor (0 to 4 stars). The Cochrane Risk of Bias (RoB 2) tool [14] was used to assess RCTs. This tool evaluates five specific domains: bias arising from the randomization process, bias due to deviations from intended interventions, bias due to missing outcome data, bias in outcome measurement, and bias in the selection of reported results. Two reviewers independently assessed each study for justifications of the risk of bias judgments with differences resolved through discussion.

### Statistical analysis

The analysis was conducted for eligible studies using R (version 4.4.2) with the meta package [15]. Due to the pooling of a mixed-design body of evidence (high-risk RCTs alongside observational studies), clinical and methodological heterogeneity was anticipated a priori. Therefore, a random-effects model with the inverse-variance method was used universally for all outcomes, with between-study variance (τ²) estimated using the DerSimonian-Laird method. Binary outcomes were pooled using risk ratios (RRs) and continuous outcomes were pooled using mean differences (MDs). Each estimate was reported along with corresponding 95% confidence intervals (CIs). We quantified the heterogeneity using the I² statistic and the chi-square test, where a p-value of < 0.1 for the chi-square test was considered significant. An I² value > 50% was considered to indicate substantial heterogeneity. Finally, we did not assess publication bias because Egger’s test is only performed when 10 or more studies are included for a particular outcome. Because of the pooling of a mixed-design body of evidence (randomized controlled trials alongside observational studies), clinical and methodological heterogeneity was anticipated a priori. A random-effects model with the inverse-variance method was therefore used for all outcomes, with between-study variance (τ²) estimated using the DerSimonian–Laird method. Although more recent methodological guidance favours restricted maximum likelihood (REML) or Paule–Mandel estimators, preferably combined with the Hartung–Knapp adjustment, particularly when the number of studies is small, we retained the DerSimonian–Laird estimator for two reasons. First, it remains the default and most widely implemented estimator in the meta package in R and in the majority of published surgical meta-analyses, facilitating direct comparability with prior reviews in this field. Second, several of our principal outcomes were informed by only three to six studies; under these sparse-data conditions the practical differences between DerSimonian–Laird and alternative estimators are often modest, while the Hartung–Knapp adjustment can itself become unstable. We acknowledge, however, that DerSimonian–Laird may underestimate between-study variance and produce overly narrow confidence intervals when k is small and heterogeneity is present. This potential underestimation of uncertainty is reflected in our GRADE assessments through the imprecision domain for outcomes with limited study numbers.

### Assessment of certainty of evidence

The certainty of evidence for each outcome was assessed using the Grading of Recommendations Assessment, Development, and Evaluation (GRADE) approach [16]. We evaluated five domains: risk of bias, inconsistency, indirectness, imprecision, and publication bias. Summary of Findings (SoF) tables were generated using GRADEpro GDT software [17].

## Results

### Study selection and characteristics

A total of 562 records were retrieved from six databases. After removing 171 duplicates, 391 records remained for screening. 28 papers were evaluated in full text, of which 11 studies fulfilled the inclusion criteria and were included in the final analysis. The study selection process, including reasons for exclusion, is illustrated in the PRISMA flow diagram in (Supplementary Figure S2). The current meta-analysis synthesizes evidence from 11 independent studies, A total of 2203 patients were evaluated, with 897 patients allocated to the indocyanine green intervention and 1306 patients as control group. Comprehensive overviews of the methodological architectures alongside the baseline clinico-demographic profiles of the respective patient cohorts are delineated in Tables 1 and 2, respectively.

**Table 1:** Summary of characteristics and methodology of included studies

Study ID	Country	Study design	Number of centers	Total sample size	ICG group (*n*)	Control group (*n*)	Indication for surgery	Type of thyroidectomy	Follow Up duration	Study period
Barreira 2024	Brazil	Retrospective cohort study	4	200	100	100	Thyroid cancer (papillary thyroid carcinoma)	Total thyroidectomy	6 months	March 2021 – March 2023
Di Lorenzo 2024	France	Retrospective cohort study	single center	131	84 (45 NIRAF alone group) and (39 NIRAF + ICG group)	47	Thyroid carcinoma	Total thyroidectomy and central neck lymph node dissection	Up to 6 months	January 2019 to March 2023
Grubnik 2023	Ukraine	Randomized controlled trial	Single center	92	44	48	Multinodular goiter (*n* = 42) thyroid cancer (*n* = 43) Graves’ disease (*n* = 7)	Total thyroidectomy	Up to 6 months	April 1, 2017 to January 1, 2022
Hassan 2025	United Arab Emirates	Retrospective matched cohort study	Single center	312	156	156	Thyroid disorders (benign and malignant)	Total thyroidectomy	Postoperative day 1	January 2022 to March 2024
Lahiri 2025	India	Retrospective-prospective cohort study	Single center	113	57	56	Thyroid disorders (mostly malignant)	Total thyroidectomy + central compartment dissection	Postoperative day 1	Nov 2022 – Dec 2023 (ICG) 2021 (Control)
Lau 2025	Australia	Retrospective cohort study	Single center	171	17	154	Benign and malignant thyroid disease	Hemi and Total thyroidectomy	not clearly defined	2019–2024
Michaelsen 2025	Denmark	Non-randomized Clinical controlled trial	single center	616	154	462	Compressive symptoms /Hyperthyroidism/ Diagnosed or suspected cancer	Total thyroidectomy	12 months	Intervention group: October 2021 to October 2023 and Control group: January 2011 to December 2020
Parfentiev 2021	Ukraine	Randomized controlled trial	Single center	58	30	28	Multinodular goiter (42), Thyroid cancer (11), Graves’ disease (5)	Total thyroidectomy	Up to 6 months	April 1, 2017 to September 10, 2019
Rossi 2023	Italy	Randomized prospective study	Single center	200	100	100	Papillary thyroid carcinoma, micropapillary thyroid carcinoma, multinodular goiter, Graves’ disease, toxic goiter	Total thyroidectomy	6 months	February 2021 – June 2021
Tae 2025	South Korea	Retrospective study	Single center	130	65	65	Papillary thyroid carcinoma	Lobectomy and total thyroidectomy	Up to 6 months	ICG group: August 2022 to March 2023 and Control group: June 2021 to July 2022
Yin 2022	China	Randomized controlled trial	Single center	180	90	90	Papillary thyroid cancer	Total thyroidectomy with unilateral or bilateral central lymph node dissection	Up to 6 months	May 2021 to January 2022

**Table 2:** Baseline demographics and clinical characteristics

Study ID	Age ICG (Mean ± SD)	Age Control (Mean ± SD)	Female ICG (%)	Female Control (%)	Malignant Cases (%)	Total Thyroidectomy (%)
Abdelrahim 2022	NR	NR	NR	NR	NR	100%
Barreira 2024	42.4 ± 11.8	47.0 ± 12.8	68%	74%	100%	100%
Devgan 2024	43.6 ± 12.6	NR	82.35%		55%	77%
Di Lorenzo 2024	51.8 ± 13.7	50.3 ± 16.1	69.23%	72.34%	100%	100%
Grubnik 2023	47.2 ± 6.4	44.6 ± 5.6	72.72%	68.75%	ICG: 47.73%, CONTROL: 45.8%	100%
Hassan 2025	43.7 ± 9.2	43.6 ± 9.4	85.30%	85.30%	NR	NR
Lahiri 2025	43.67 ± 41.07	33.67 ± 59.35	50.88%	62.50%	CONTROL: 96.43%, ICG: 94.74%	CONTROL: 71.43%, ICG: 71.93%
Lau 2025	51.0 ± 12.4	51.9 ± 15.6	76	73	NR	24.56%
Michaelsen 2025	55 ± 18.71	53.67 ± 17.84	70.80%	72.50%	ICG GROUP: 51.3%, Control group: 51.3%	ICG GROUP: 57.1%, Control group: 57.1%
Neagoe 2024	50.1 ± 19.1	49.7 ± 18.2	79.60%	82.10%	ICG GROUP: 10.7%%, control group :18.9%	100%
Oravec 2026	NR	NR	NR	NR	77.10%	68.60%
Parfentiev 2021	44.38 ± 11.22	43.24 ± 12.15	64.28%	53.33%	ICG Group: Thyroid cancer: 4 N, Control Group: 7 N	100%
Rossi 2023	44.52 ± 11.22	44.82 ± 10.13	78%	84%	ICG Group: Papillary thyroid carcinoma 48%, Micropapillary thyroid carcinoma 15%, in control Group: Papillary thyroid carcinoma: 43%, Micropapillary thyroid carcinoma: 10%	100%
Tae 2025	43.1 ± 11.6	39.7 ± 12.1	86.20%	83.10%	100%	40%
Yin 2022	42.1 ± 9.5	42.7 ± 9.1	77.80%	82.20%	100%	100%

### Risk of bias assessment

The Four randomized trials (evaluated using RoB 2) were judged, in which (Rossi et al., 2023 and Yin et al., 2022) were judged to be at low risk of bias and (Grubnik et al., 2023 and Parfentiev et al., 2021) were judged to shows some concerns primarily (Supplementary Figure S1). The Cohort and non-randomized studies (evaluated with the NOS) were rated as good quality for the cohort study (9 stars) and poor quality for (Lahiri et al., 2025 and Lau et al., 2025) (7 & 6 stars respectively) (Supplementary Table S2).

### Clinical and visualization outcomes

#### Operative time

**Fig. 1:**
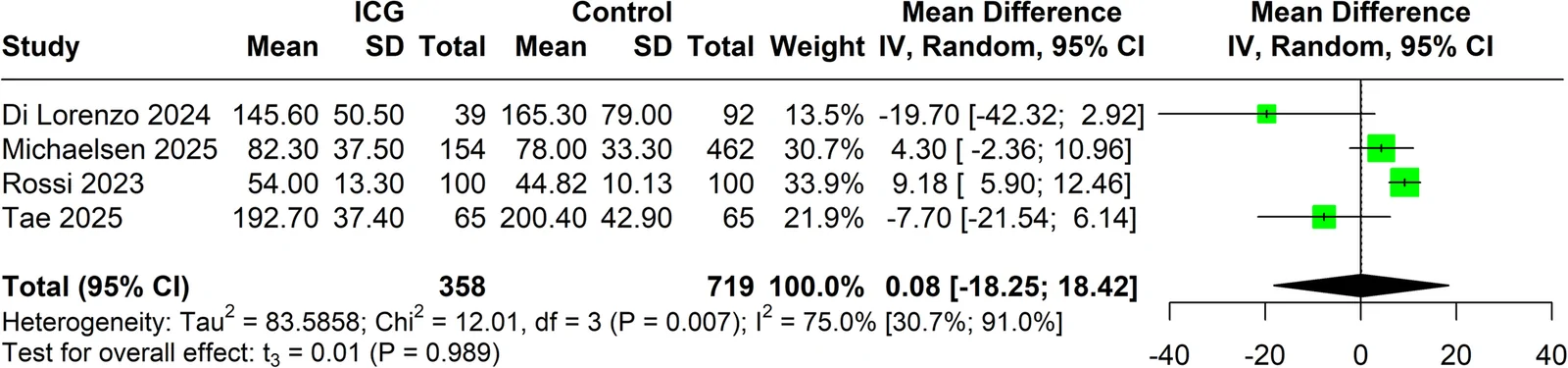
Forest Plot of Operative time

The pooled analysis of four studies including 1,077 patients showed no significant difference in operative time between ICG and Control groups (MD 0.08, 95% CI − 18.25 to 18.42; p = 0.989), Results were heterogeneous (I²=75%) (Fig. 1).

#### Transient hypocalcemia

**Fig. 2:**
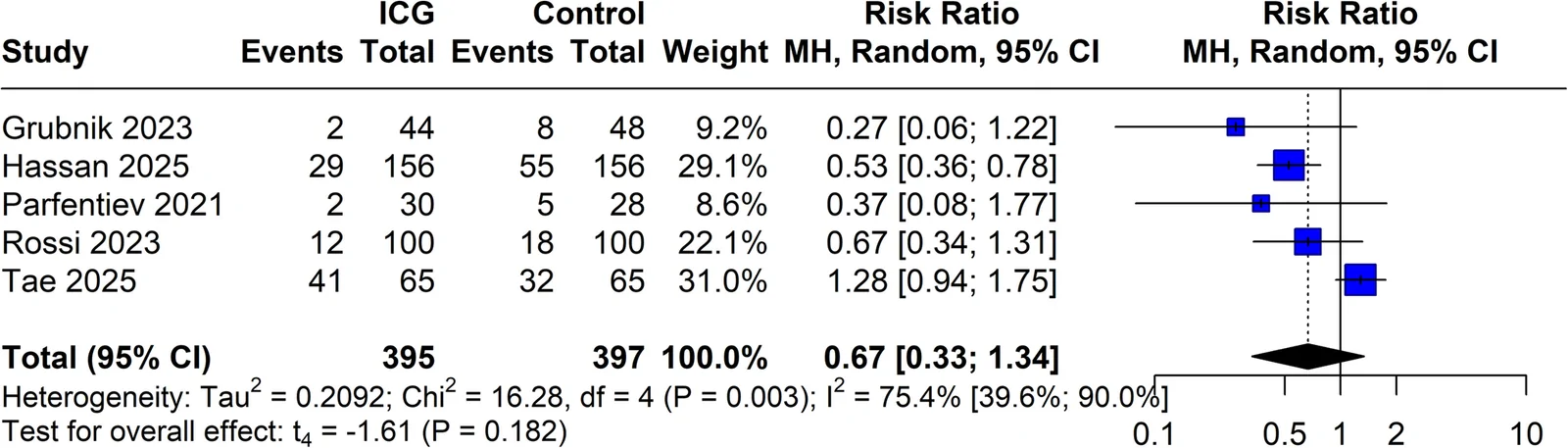
Forest Plot of Transient hypocalcemia

**Fig. 3:**
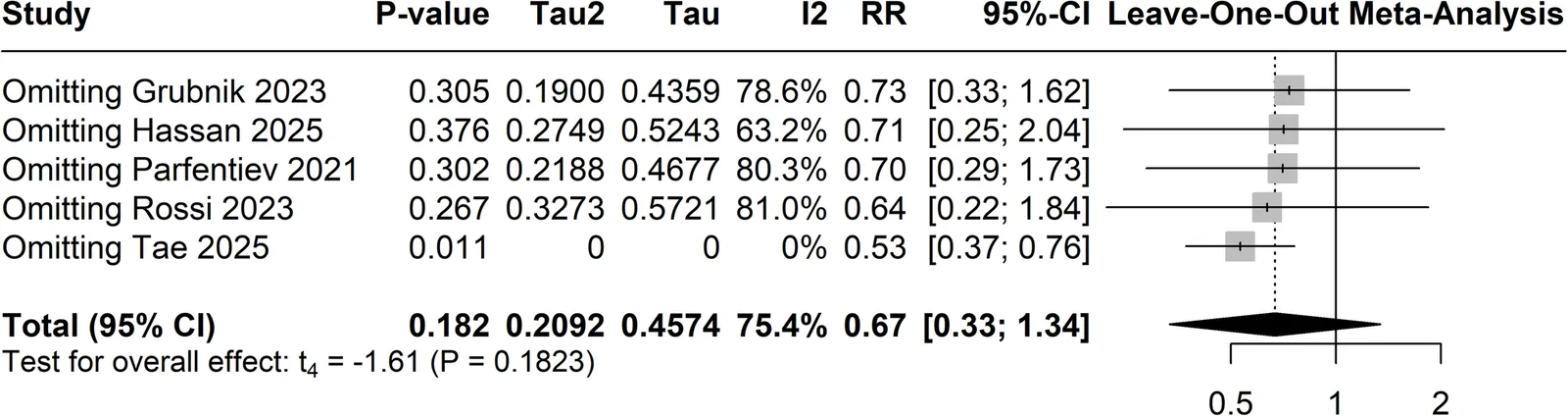
Forest Plot of Transient hypocalcemia LOO

The pooled analysis of five studies including 792 patients showed no significant difference in transient hypocalcemia between ICG and Control groups (RR 0.67, 95% CI 0.33–1.34; p = 0.182), Results were heterogeneous (I²=75.4%) (Fig. 2). Leave-one-out sensitivity analysis indicated that exclusion of the Tae 2025 study reduced heterogeneity to 0% and rendered the result statistically significant in favor of ICG (RR 0.53, 95% CI 0.37–0.76) (Fig. 3).

#### Permanent hypocalcemia

**Fig. 4:**
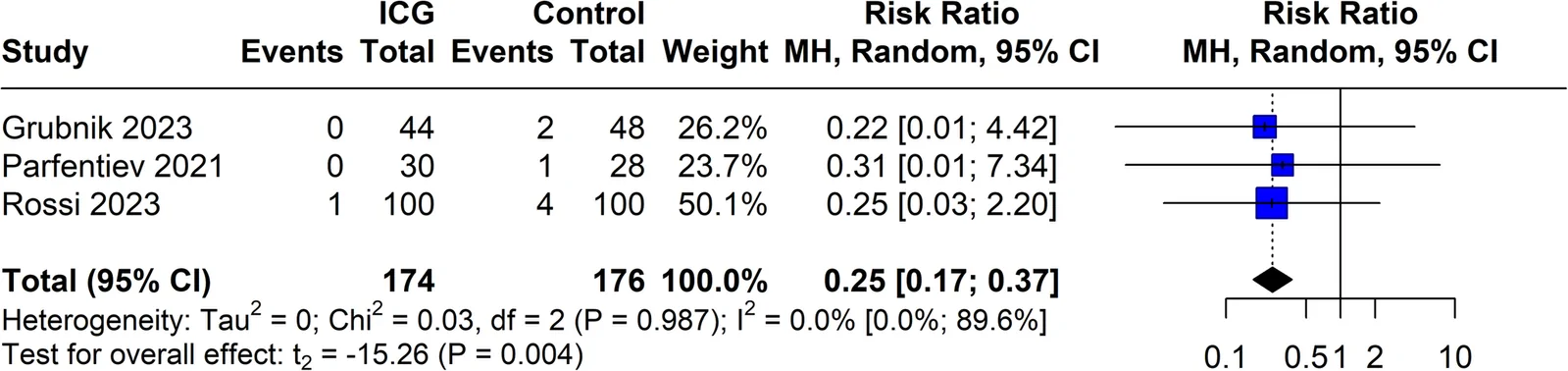
Forest Plot of Permanent Hypocalcemia

The pooled analysis of three studies including 350 patients showed significantly lower permanent hypocalcemia in the ICG group compared to Control (RR 0.25, 95% CI 0.17-0.37; p=0.004), Results were homogeneous (I²=0%) (Fig. 4).

#### Transient hypoparathyroidism

**Fig. 5:**
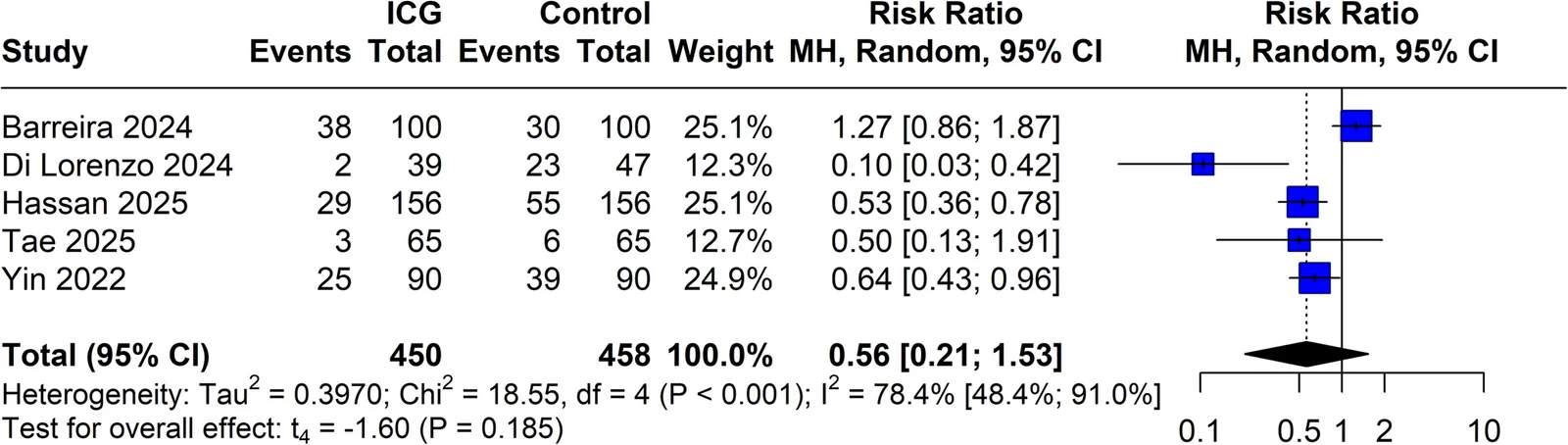
Forest Plot of Transient hypoparathyroidism

**Fig. 6:**
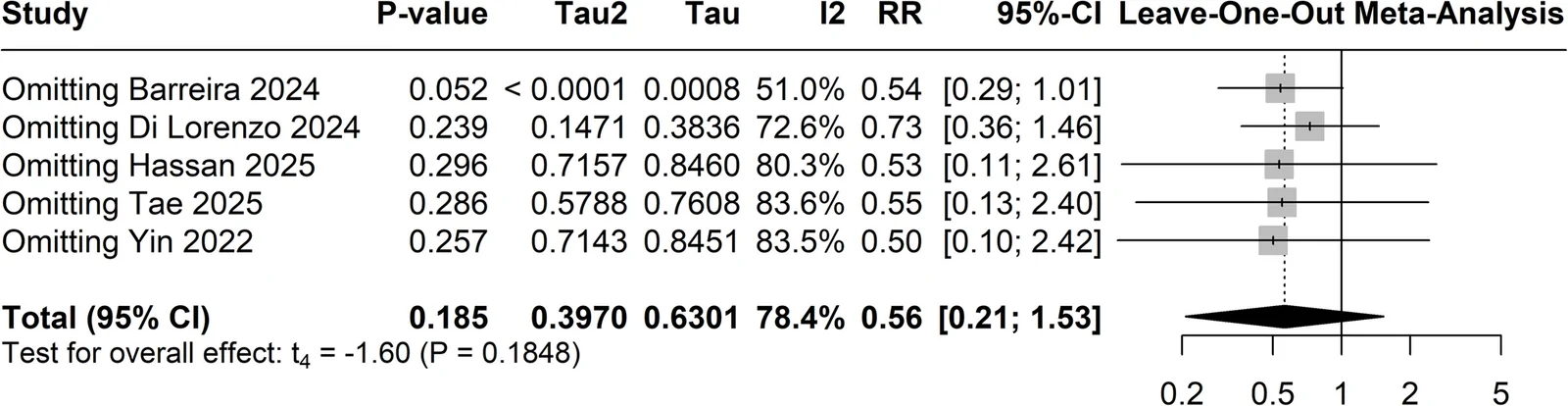
Forest Plot of Transient hypoparathyroidism LOO

The pooled analysis of five studies including 908 patients showed no significant difference in transient hypoparathyroidism between ICG and Control groups (RR 0.56, 95% CI 0.21-1.53; p=0.185), Results were heterogeneous (I²=78.4%) (Figure 5). Leave-one-out sensitivity analysis demonstrated that no single study, when omitted, resolved the heterogeneity or rendered the overall effect statistically significant, indicating that the instability is distributed across studies rather than driven by any one contributor (Fig. 6).

#### Permanent hypoparathyroidism

**Fig. 7:**
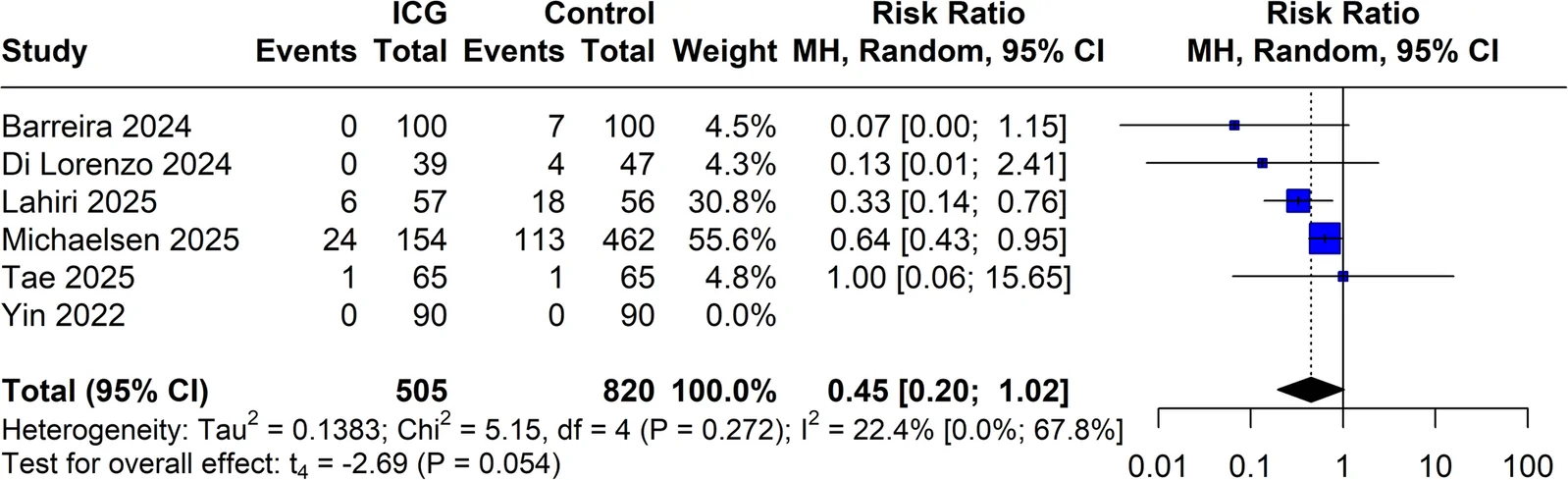
Forest Plot of Permanent hypoparathyroidism

**Fig. 8:**
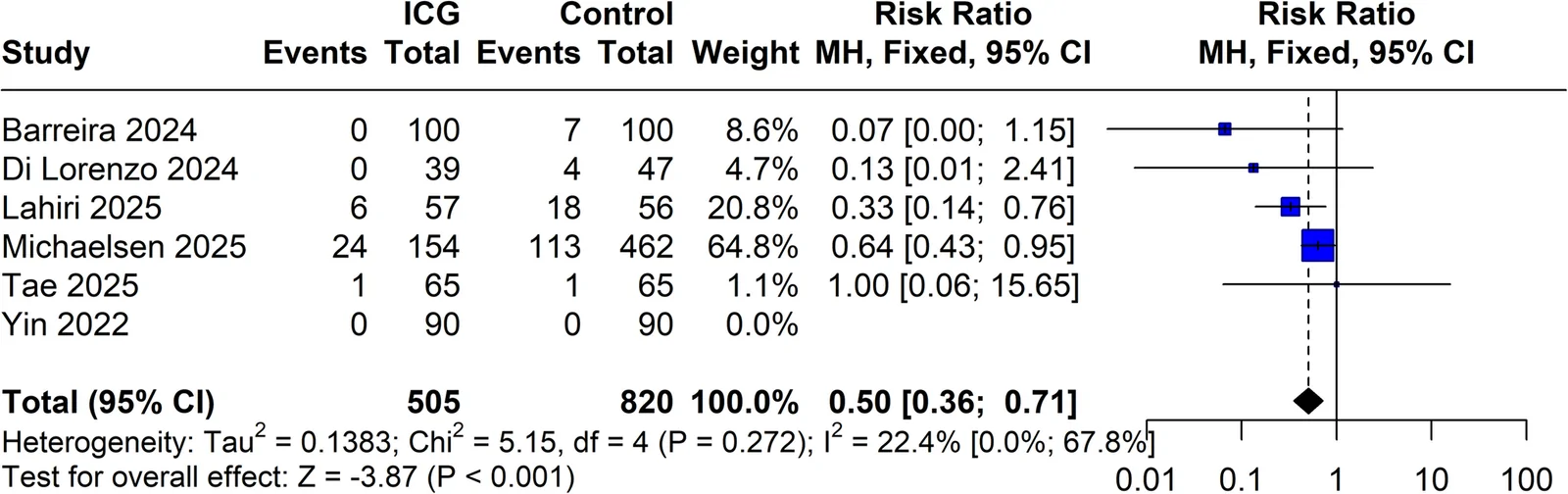
Forest Plot of Permanent hypoparathyroidism (Fixed-effect sensitivity analysis)

The pooled analysis of six studies including 1,325 patients showed a non-significant trend toward reduced permanent hypoparathyroidism in the ICG group under our pre-specified random-effects model (RR 0.45, 95% CI 0.20–1.02; p=0.054), with acceptable heterogeneity (I²=22.4%) (Fig. 7). A sensitivity analysis using a fixed-effect model yielded a statistically significant result (RR 0.50, 95% CI 0.36–0.71; p<0.001), with low heterogeneity (Fig. 8). This fixed-effect estimate is presented strictly as a sensitivity analysis and should not be interpreted as evidence of a statistically significant effect for this outcome, given that our pre-specified primary model did not reach significance; the discrepancy likely reflects the sensitivity of significance testing to model assumptions when the point estimate is meaningfully below the null but the confidence interval remains wide, rather than a genuine change in the underlying evidence.

#### Inadvertent parathyroid removal

**Fig. 9:**
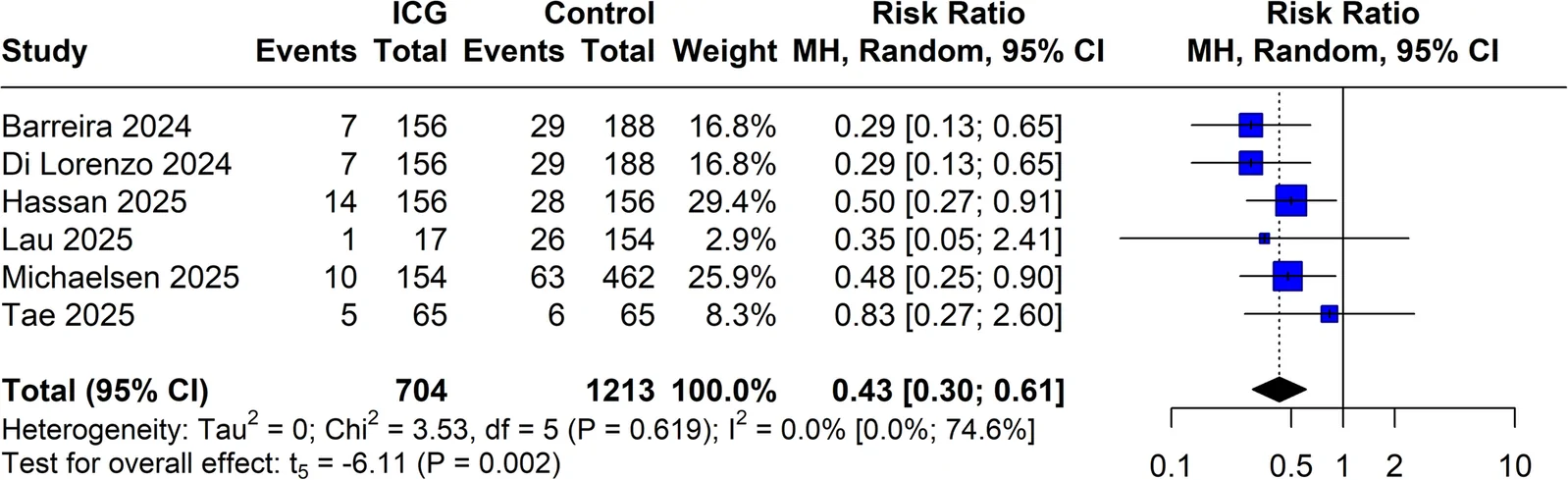
Forest Plot of Inadvertent parathyroid removal

**Fig. 10:**
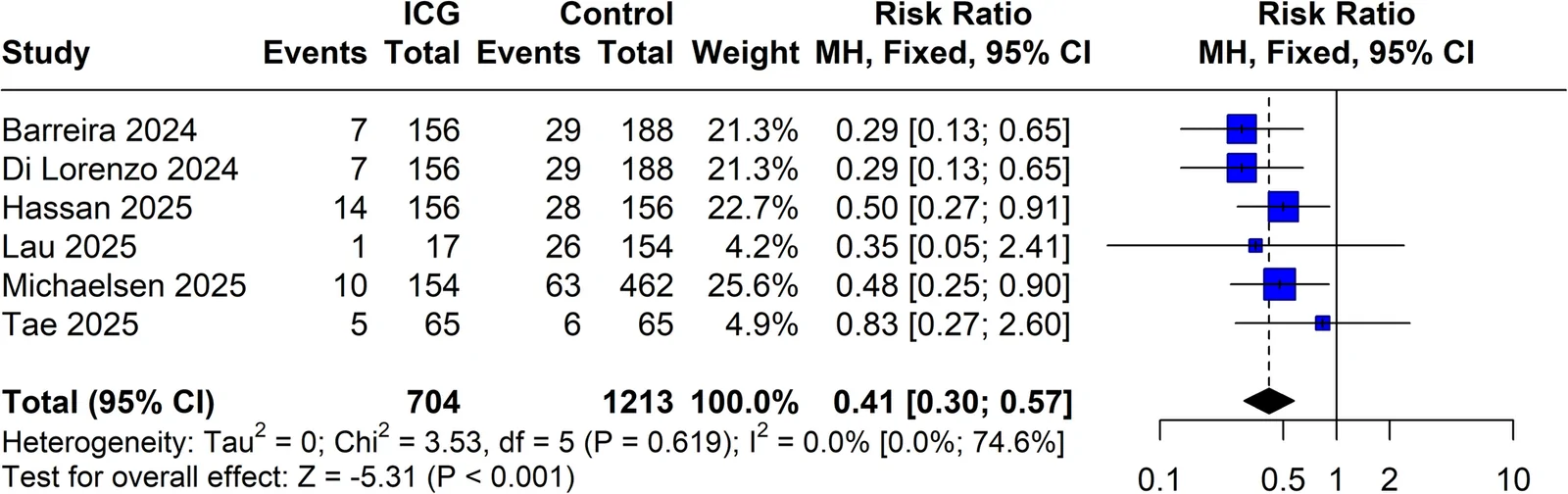
Forest Plot of Inadvertent parathyroid removal (Fixed-effect sensitivity analysis)

The pooled analysis of six studies including 1,917 patients showed a significant reduction in inadvertent parathyroid removal in the ICG group compared to controls (RR 0.43, 95% CI 0.30–0.61; p=0.002) under random-effects model and Results were homogeneous (I²=0.0%) (Fig. 9). Fixed-effect sensitivity analysis yielded a consistent result (RR 0.41, 95% CI 0.30–0.57; P < 0.001) (Fig. 10).

#### Postoperative parathyroid hormone (PTH) levels

**Fig. 11:**
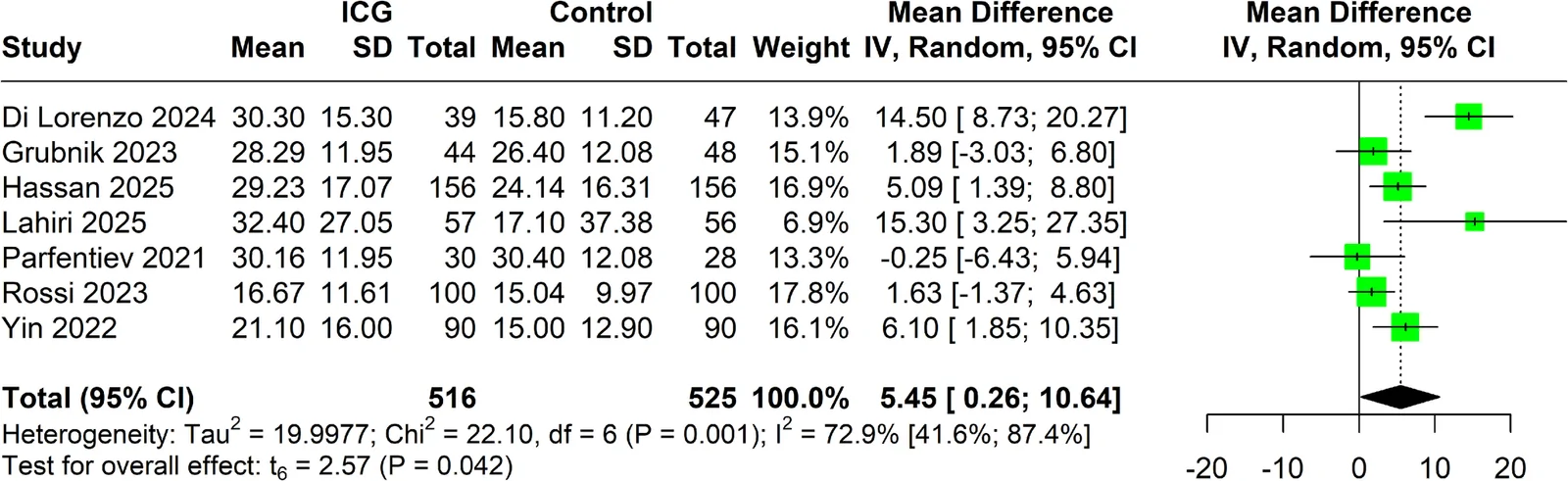
Forest Plot of PTH (pg/mL)

The pooled analysis of seven studies including 1,041 patients showed a significantly higher postoperative PTH level in the ICG group compared to controls (MD 5.45 pg/mL, 95% CI 0.26–10.64; p=0.042), indicating better parathyroid functional preservation with ICG guidance (Fig. 11). The pooled mean difference was 5.45 pg/mL (95% CI 0.26–10.64). Although the point estimate reached statistical significance, the lower bound of the confidence interval approaches the null, and the absolute increment is modest relative to typical postoperative PTH ranges. We have therefore revised the text to emphasize that this difference should be viewed as supportive evidence of better functional parathyroid preservation rather than as a large, independently clinically meaningful effect. The moderate GRADE certainty rating already incorporated this degree of imprecision.

#### Postoperative serum calcium levels

**Fig. 12:**
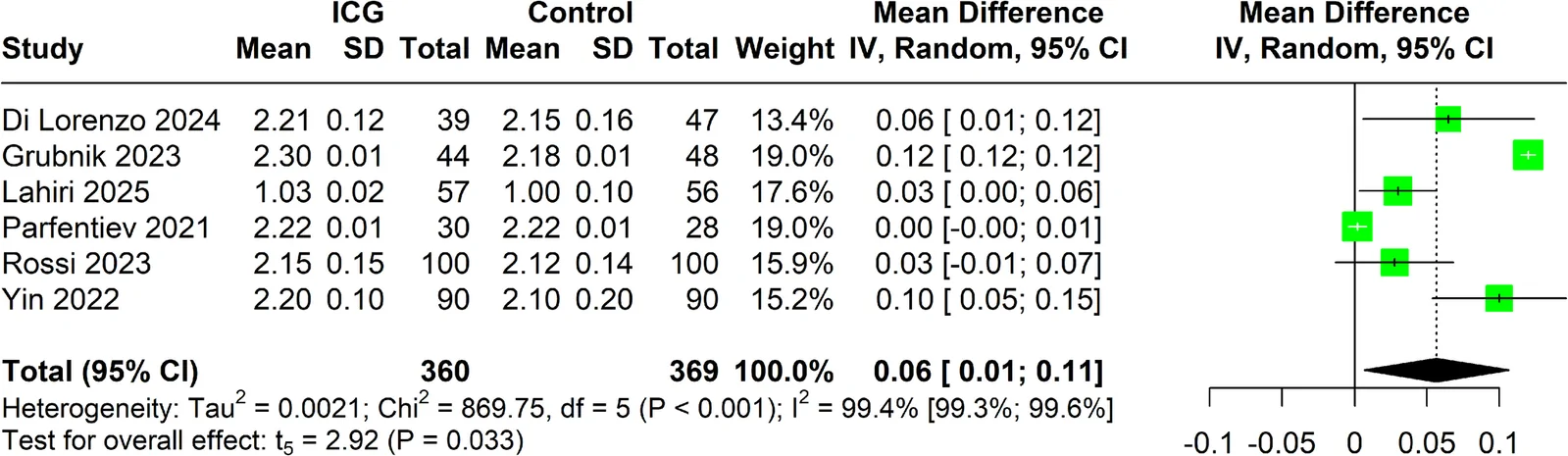
Forest Plot of Calcium level (mmol/L)

The pooled analysis of six studies including 729 patients showed a significantly higher postoperative calcium level in the ICG group compared to controls (MD 0.06 mmol/L, 95% CI 0.01–0.11; p=0.033), suggesting better calcium homeostasis preservation with ICG guidance (Fig. 12). The pooled mean difference was 0.06 mmol/L (95% CI 0.01–0.11). While statistically significant, this absolute difference is small. We now explicitly state in both the Results and Discussion that an increment of this magnitude is unlikely, on its own, to alter clinical decision-making or reduce the need for calcium supplementation in most patients. The finding is best interpreted as a biochemical signal of improved parathyroid preservation rather than as a clinically decisive change in calcium homeostasis. This interpretation is consistent with the moderate certainty rating already assigned under GRADE, which reflected imprecision around a small absolute effect.

Substantial statistical heterogeneity was observed for both transient hypocalcemia (I² = 75.4%) and transient hypoparathyroidism (I² = 78.4%). Leave-one-out sensitivity analysis identified the Tae 2025 study as the principal contributor to heterogeneity in transient hypocalcemia; its exclusion reduced I² to 0% and rendered the pooled estimate statistically significant. No single study accounted for the heterogeneity in transient hypoparathyroidism. Although formal subgroup or meta-regression analyses were not performed (owing to the limited number of studies contributing to each outcome), important study-level differences that may plausibly explain the observed variability include: extent of thyroidectomy (total versus less-than-total), performance of central neck dissection, proportion of malignant versus benign disease, surgeon experience and institutional volume, specific fluorescence imaging platform, ICG dosing and injection protocol, timing of fluorescence assessment relative to gland dissection and differing biochemical thresholds and time points used to define “transient” hypocalcemia or hypoparathyroidism.

### GRADE assessment

The certainty of evidence for each of the eight outcomes was assessed according to the five GRADE domains (risk of bias, inconsistency, indirectness, imprecision and publication bias) using GRADEpro GDT software [17]. In accordance with standard GRADE guidance, evidence derived predominantly from randomized controlled trials began at high certainty, while evidence derived predominantly from observational studies began at low certainty. When both study designs contributed to a pooled estimate, the starting level was determined by the design that contributed the majority of the weight or the majority of the studies informing that outcome; this decision is documented for each endpoint in the footnotes of the Summary of Findings table (Supplementary Table S3). Certainty was then modified through the standard process of downgrading (and, where applicable, upgrading) according to the domain-specific considerations detailed below. No outcome was upgraded. The complete Summary of Findings table, including per-outcome footnotes explaining the starting level and all subsequent rating decisions, is provided in (Supplementary Table S3).

#### Inadvertent parathyroid removal (high certainty)

No downgrading was applied. Risk of bias was not serious, as the pooled estimate was consistent across studies rated at low risk of bias (RoB 2) and good quality (NOS); inconsistency was not serious (I²=0.0%); indirectness was not serious, given comparable surgical populations and outcome definitions across studies; imprecision was not serious, as the 95% CI was narrow and excluded the null (RR 0.43, 95% CI 0.30–0.61) across a relatively large sample (1,917 patients); publication bias was not suspected, though not formally tested.

#### Permanent hypocalcemia (moderate certainty)

Downgraded one level for imprecision, owing to the relatively small number of contributing studies (n=3) and total events despite the narrow confidence interval (RR 0.25, 95% CI 0.17–0.37; 350 patients), falling short of the optimal information size typically required for high certainty. Risk of bias and inconsistency were not serious (I²=0%); indirectness was not serious.

#### Transient hypoparathyroidism (very low certainty)

Downgraded one level for risk of bias (unblinded outcome assessment in contributing RCTs), one level for inconsistency (I²=78.4%, unresolved by leave-one-out sensitivity analysis), and one level for imprecision (wide 95% CI crossing the null, RR 0.56, 95% CI 0.21–1.53). The cumulative effect of serious concerns across three domains warranted a three-level downgrade to very low certainty.

#### Transient hypocalcemia (Low certainty)

Downgraded one level for inconsistency (I²=75.4%), attributable predominantly to a single study (Tae 2025), whose exclusion resolved heterogeneity and rendered the effect significant on leave-one-out sensitivity analysis, and one level for imprecision (95% CI crossing the null, RR 0.67, 95% CI 0.33–1.34). Risk of bias was not rated as an additional separate downgrade to avoid double-counting concerns already reflected in the inconsistency rating.

#### Postoperative parathyroid hormone (PTH) levels (moderate certainty)

Downgraded one level for imprecision, as the 95% CI approached the null at its lower bound despite reaching statistical significance (MD 5.45 pg/mL, 95% CI 0.26–10.64; p=0.042), indicating a degree of uncertainty around the true magnitude of benefit. Risk of bias, inconsistency, and indirectness were not serious.

#### Postoperative serum calcium levels (moderate certainty)

Downgraded one level for imprecision, reflecting the small absolute magnitude of the mean difference relative to its confidence interval (MD 0.06 mmol/L, 95% CI 0.01–0.11; p=0.033), raising uncertainty as to the clinical (rather than statistical) significance of the observed difference. Risk of bias, inconsistency, and indirectness were not serious.

#### Operative time (low certainty)

Downgraded one level for inconsistency (I²=75%) and one level for imprecision, given the very wide confidence interval spanning both clinically important benefit and harm (MD 0.08 min, 95% CI −18.25 to 18.42; p=0.989).

Consideration was also given to upgrading the certainty of evidence for outcomes informed substantially by observational studies, per GRADE criteria permitting upgrading in the presence of a large magnitude of effect, a dose-response gradient, or plausible confounding that would be expected to reduce rather than create the observed effect. No outcome in this review was upgraded on these grounds. While several point estimates were of considerable magnitude (e.g., permanent hypocalcemia, RR 0.25; inadvertent parathyroid removal, RR 0.43), a dose-response relationship could not be evaluated given inconsistent reporting of ICG dosing across the included studies, and plausible sources of confounding in the observational evidence (e.g., differential surgeon experience or case selection favoring ICG use in more straightforward cases) could not be confidently assumed to bias against the observed effect. Accordingly, all certainty ratings presented reflect downgrading only from the applicable initial rating, with no upgrading applied.

Publication bias was considered qualitatively for every outcome as one of the five GRADE domains. Formal statistical assessment (such as Egger’s regression test or funnel-plot asymmetry) was not performed because fewer than ten studies contributed to each outcome, rendering such tests unreliable. In the absence of formal testing, we evaluated the potential for publication bias on the basis of the following qualitative criteria: (i) the relatively small number of available studies, (ii) the mixture of randomized and observational designs, (iii) the absence of registered but unpublished trials identified in ClinicalTrials.gov that reported opposing results, and (iv) the consistency of effect direction across the included studies. For all eight outcomes we judged publication bias to be undetected. Consequently, no outcome was downgraded for publication bias. This judgement is recorded in the footnotes of the Summary of Findings table (Supplementary Table S3).

## Discussion

Our updated meta-analysis provides evidence that the integration of indocyanine green (ICG) fluorescence with near-infrared (NIR) imaging is associated with benefit for select outcomes in thyroid surgery, particularly inadvertent parathyroid removal and permanent hypocalcemia, both supported by high-to-moderate certainty evidence. However, findings for other clinically important outcomes, including permanent hypoparathyroidism, transient hypoparathyroidism, and transient hypocalcemia, were non-significant or of low-to-very-low certainty, and should not be interpreted as establishing a uniform clinical benefit of ICG-guided imaging across all parathyroid-related endpoints. The observed reduction in inadvertent parathyroid excision and permanent hypocalcemia is nonetheless statistically robust and clinically relevant, given the long-term morbidity associated with permanent parathyroid failure [18].

A key strength of ICG-guided imaging lies in its ability to bridge a long-standing limitation in thyroid surgery: the disconnect between anatomical identification and functional viability of the parathyroid glands [19]. While conventional visual inspection allows surgeons to identify suspected parathyroid tissue, it provides little to no information regarding vascular integrity. In contrast, ICG angiography enables real-time perfusion assessment, allowing intraoperative discrimination between well-vascularized glands and those at risk of ischemic injury. This functional insight likely explains the observed improvement in postoperative PTH and calcium levels in the ICG group [10].

Interestingly, the primary pre-specified random-effects analysis for permanent hypoparathyroidism did not reach statistical significance (RR 0.45, 95% CI 0.20–1.02; *p* = 0.054). A post-hoc fixed-effect sensitivity analysis yielded a statistically significant result (RR 0.50, 95% CI 0.36–0.71; *p* < 0.001); however, this finding must be interpreted strictly as a sensitivity analysis and does not override the primary random-effects result. Consequently, the current evidence remains insufficient to demonstrate a definitive reduction in permanent hypoparathyroidism with ICG-guided imaging. While the point estimate lies in a favorable direction, the confidence interval crosses the null and the certainty of evidence is rated low. Claims of benefit for this specific endpoint should therefore be avoided until more precise data become available.

The pattern of results suggests that any potential benefit of ICG guidance may be more evident for permanent hypocalcemia and inadvertent parathyroid removal than for permanent hypoparathyroidism, for which the primary analysis remains inconclusive. Transient hypocalcemia and hypoparathyroidism are multifactorial phenomena, often influenced by temporary ischemia, surgical manipulation, or postoperative inflammatory changes as factors that may not be fully preventable even with optimal visualization. This may also explain the substantial heterogeneity observed in transient outcomes across included studies, where differences in biochemical thresholds, timing of assessment, and surgical extent likely contributed to variability [20].

The stability of findings related to permanent hypocalcemia and inadvertent gland removal, both of which demonstrated low heterogeneity, reinforces the reliability of these two outcomes specifically as indicators of true surgical benefit. Moreover, the consistency of effect estimates across sensitivity analyses supports the robustness of our conclusions for these outcomes in particular, though this stability does not extend uniformly to the remaining endpoints assessed, where heterogeneity was substantial and effect estimates less consistent.

Our findings are broadly consistent with several recently published meta-analyses evaluating fluorescence-guided parathyroid preservation. Canali et al. (2025) [7], restricting their analysis to randomized controlled trials of camera-based NIR autofluorescence, similarly reported reduced rates of transient hypocalcemia and hypoparathyroidism with fluorescence guidance compared with visual identification alone, supporting the direction of effect observed in our review despite differences in study design inclusion criteria. Cheung et al. (2026) [21], pooling 28 studies of both autofluorescence and ICG-based fluorescent imaging techniques, likewise reported significantly improved parathyroid gland identification rates and reduced incidental parathyroidectomy and short-term hypoparathyroidism with fluorescence guidance, findings that parallel our own significant reduction in inadvertent parathyroid removal. Notably, however, Cheung et al. reported a significant reduction specifically in short-term (rather than permanent) hypoparathyroidism, whereas our analysis found the opposite pattern: a significant reduction in permanent hypocalcemia but only a non-significant trend for permanent hypoparathyroidism, with transient outcomes remaining non-significant in our pooled estimates. This divergence may reflect differences in outcome stratification (time-based thresholds for “short-term” versus formal transient/permanent classification), the specific fluorescence modalities pooled (NIRAF alone versus combined NIRAF/ICG cohorts), or the inclusion of observational alongside randomized evidence in our review. Javed et al. (2025) [22], restricting their synthesis to RCTs of NIRAF specifically, reported effect directions consistent with our findings but similarly cautioned that heterogeneity in surgical and imaging protocols limited the precision of pooled estimates, a limitation we also identify in our own review. Taken together, this comparison suggests our findings are directionally consistent with the broader recent literature, while also highlighting those specific parameters namely outcome timepoint definitions and specific imaging modality that may drive some inter-review variability in reported effect estimates.

From a practical perspective, the lack of increase in operative time is a further finding of note, suggesting that adoption of ICG imaging does not introduce additional procedural complexity once the learning curve is overcome; however, this finding was itself associated with substantial heterogeneity (I²=75%) and was rated low certainty, and should therefore be interpreted as reassuring rather than definitive. This is particularly relevant in high-volume endocrine surgery settings, where efficiency and reproducibility are critical. Additionally, the technique’s ability to provide on-demand intraoperative feedback without reliance on preoperative preparation enhances its feasibility in routine clinical practice [21].

Several limitations must be acknowledged when interpreting these findings. First, the included body of evidence comprises a mixture of randomized (*n* = 4) and observational (*n* = 7) studies, with inherent variability in study design, patient selection, and surgical expertise. The pooling of these designs in a single analysis, while necessary given the limited number of RCTs available for several outcomes, means that pooled estimates may be influenced by residual confounding inherent to non-randomized designs; this concern is partly mitigated by our random-effects modeling approach and is reflected in our GRADE risk-of-bias downgrading for affected outcomes, but readers should nonetheless interpret estimates driven predominantly by observational data with appropriate caution. The absence of blinding in most trials further introduces potential performance and detection bias, particularly for subjectively assessed outcomes.

Second, Substantial statistical heterogeneity was present for the transient outcomes (I² = 75.4% for transient hypocalcemia and 78.4% for transient hypoparathyroidism). Although leave-one-out analyses were performed, formal subgroup analyses or meta-regression exploring clinically relevant effect modifiers were not undertaken because the number of studies contributing to each outcome (five) is insufficient to support statistically reliable stratification without a high risk of spurious findings. Nevertheless, several study-level differences that could plausibly account for the observed variability deserve explicit recognition. These include variation in the extent of thyroidectomy and the performance of central compartment lymphadenectomy, the proportion of patients with malignant versus benign disease, differences in surgeon experience and institutional case volume, the specific near-infrared imaging platform employed, heterogeneity in ICG dosing regimens and injection timing, the precise moment at which fluorescence assessment was performed relative to parathyroid dissection, and, importantly, inconsistent biochemical definitions and postoperative time windows used to classify hypocalcemia and hypoparathyroidism as “transient.” While permanent outcomes demonstrated far lower heterogeneity (I² = 0% and 22.4%), consistent with a more uniform conceptual definition (ongoing need for supplementation at approximately 12 months), the transient endpoints appear particularly sensitive to these methodological and clinical differences. A descriptive summary of the key study characteristics that may drive this heterogeneity is provided in (Supplementary Table S4).

Third, several outcomes, particularly permanent hypocalcemia (three studies, 350 patients), were based on a relatively small number of studies and limited event numbers, which may affect the stability and precision of pooled estimates derived from standard inverse-variance random-effects modeling; alternative methods better suited to sparse-event data were not applied in the current review. We did not calculate prediction intervals or formally test for small-study effects or publication bias, given the insufficient number of studies (< 10) across all outcomes for reliable application of these methods; our leave-one-out sensitivity analyses served as our primary approach to influence diagnostics in their place. In addition, between-study variance was estimated with the DerSimonian–Laird method. While this estimator is widely used and was chosen for consistency with the majority of published meta-analyses in endocrine surgery, it can underestimate uncertainty when the number of studies is small (three to six studies for several outcomes in the present review). More robust alternatives such as REML or Paule–Mandel with Hartung–Knapp adjustment were not applied. Consequently, the reported confidence intervals for outcomes with sparse data may be somewhat anti-conservative; this limitation is already captured in the GRADE imprecision ratings for those endpoints.

Finally, our search was restricted to English-language publications, given resource constraints for translation and quality assessment of non-English studies, and follow-up durations across studies were often insufficient to fully capture long-term parathyroid function, particularly in borderline or late-recovery cases. Collectively, the limited number of trials, variability in follow-up duration and outcome definitions, and the heterogeneity in fluorescence technique and administration protocol temper the generalizability of our findings and highlight the need for future primary studies employing standardized definitions, protocols, and randomized designs. A key limitation is the clinical and technological heterogeneity among fluorescence modalities, including NIR autofluorescence (NIRAF), ICG angiography, and dual-modality approaches. Because these techniques differ in their mechanisms and clinical information, pooling them may introduce heterogeneity and assumes unproven clinical equivalence. However, pooling was justified by their shared goal of preventing parathyroid injury and by the limited number of studies available for each modality, which restricted reliable subgroup analyses. Future reviews with larger evidence bases should conduct modality-specific analyses to determine whether benefits are modality-specific or represent a general effect of fluorescence-guided surgery.

However formal statistical tests for publication bias were appropriately omitted because fewer than ten studies were available for each outcome, publication bias remains a GRADE domain that must be considered qualitatively. On the basis of the available evidence, we judged publication bias to be undetected for all outcomes; however, the small evidence base means that the possibility of missing negative studies cannot be entirely ruled out. This residual uncertainty is acknowledged here and was taken into account when interpreting the overall strength of the findings.

Taken as a whole, the evidence from this review supports a real and clinically meaningful benefit of ICG-guided NIR imaging for reducing inadvertent parathyroid removal and permanent hypocalcemia specifically. However, the non-significant findings for permanent hypoparathyroidism under our pre-specified random-effects model, the substantial unexplained heterogeneity in transient outcomes, and the overall certainty of evidence ranging from very low to high across endpoints collectively indicate that this technology should not yet be regarded as uniformly transformative across all aspects of parathyroid preservation. Clinicians and future guideline developers should weigh these outcome-specific certainty distinctions when considering the adoption of ICG-guided imaging in routine practice. Rather than replacing surgical expertise, this technology appears to enhance intraoperative decision-making by adding an objective layer of functional assessment for the outcomes it most reliably improves. Future research should focus on well-designed, adequately powered randomized trials with standardized imaging protocols, harmonized outcome definitions, and longer follow-up to further clarify its role, particularly in high-risk populations such as reoperative thyroid surgery or extensive lymph node dissection.

## Conclusions

Our systematic review and meta-analysis demonstrate that near-infrared autofluorescence with indocyanine green (ICG) imaging is associated with a reduction in inadvertent parathyroid removal (high certainty) and permanent hypocalcemia (moderate certainty), along with higher postoperative PTH and serum calcium levels (moderate certainty), compared with standard white-light surgery. Evidence for a reduction in permanent hypoparathyroidism was inconclusive: the primary random-effects analysis did not reach statistical significance and was rated low certainty. Current data are therefore insufficient to claim a definitive benefit for this endpoint. Operative time was comparable between groups, though this finding was limited by substantial heterogeneity (low certainty). Transient hypoparathyroidism and transient hypocalcemia showed favorable but statistically non-significant trends, both rated very low to low certainty due to substantial heterogeneity and imprecision. Given this certainty profile, ICG-guided near-infrared autofluorescence should be regarded as a promising intraoperative adjunct with the strongest evidentiary support for reducing inadvertent gland removal and permanent hypocalcemia specifically, rather than as a uniformly validated standard across all parathyroid-related outcomes. Future large-scale randomized controlled trials with standardized imaging protocols, blinded outcome assessment, and longer follow-up are needed to confirm these findings, resolve the observed heterogeneity in transient outcomes, and clarify the intervention’s role in high-risk surgical populations.

## Supplementary Information

Below is the link to the electronic supplementary material.

Supplementary Material 1

Supplementary Material 2

Supplementary Material 3

Supplementary Material 4

Supplementary Material 5

## Data Availability

No datasets were generated or analysed during the current study.

## Declarations

### Registration

This systematic review was prospectively registered with PROSPERO (ID: CRD420261348529).
