# Supplementary material for: Indocyanine green (ICG) guided thyroidectomy versus conventional thyroidectomy and the impact on parathyroid function preservation: an updated GRADE-assessed systematic review and meta-analysis
Source: Langenbecks Arch Surg. 2026 Sep 1;411(1):257. doi: 10.1007/s00423-026-04210-3 (PMC13630843; doi:10.1007/s00423-026-04210-3)
Supplement: Supplementary file 3 [file 423_2026_4210_MOESM3_ESM.docx]

***SUPPLEMENTARY MATERIALS***

# **INDEX**

| Page | Supplementary Item |
| --- | --- |
| 2 | **Supplementary Table S1.** Comprehensive literature search strategy across electronic databases |
| 3 | **Supplementary Table S2.** Quality assessment of the included observational studies utilizing the Newcastle-Ottawa Scale (NOS) |
| 4 | **Supplementary Figure S1.** Risk of bias summary for the included randomized controlled trials evaluated by the Cochrane RoB 2 tool |
| 5 | **Supplementary Figure S2.** PRISMA flow diagram |
| 6 | **Supplementary Table S3.** GRADE evidence profile for primary and secondary outcomes |

***Supplementary Table S1.*** *Comprehensive literature search strategy across electronic databases*

| **Database** | **Search build** | **Results** |
| --- | --- | --- |
| **PubMed** | (("Parathyroid Gland" OR "parathyroid") AND ("Thyroid Gland" OR "Thyroidectomy") AND ("Autofluorescence" OR "near infrared" OR "Near-Infrared" OR "NIR" OR "NIRAF" OR "fluorescence imaging" OR "Optical imaging" OR "Indocyanine Green" OR "ICG) AND ("Surgical Procedures" OR "Operative" OR "Surgery” OR "parathyroidectomy" OR "Neck surgery" OR "endocrine surgery")) [All Fields] | 2 |
| **WEB OF SCIENCE** | All Fields (("Parathyroid Gland" OR "parathyroid") AND ("Thyroid Gland" OR "Thyroidectomy") AND ("Autofluorescence" OR "near infrared" OR "Near-Infrared" OR "NIR" OR "NIRAF" OR "fluorescence imaging" OR "Optical imaging" OR "Indocyanine Green" OR "ICG) AND ("Surgical Procedures" OR "Operative" OR "Surgery” OR "parathyroidectomy" OR "Neck surgery" OR "endocrine surgery")) | 189 |
| **Scopus** | TITLE-ABS-KEY (("Parathyroid Gland" OR "parathyroid") AND ("Thyroid Gland" OR "Thyroidectomy") AND ("Autofluorescence" OR "near infrared" OR "Near-Infrared" OR "NIR" OR "NIRAF" OR "fluorescence imaging" OR "Optical imaging" OR "Indocyanine Green" OR "ICG) AND ("Surgical Procedures" OR "Operative" OR "Surgery” OR "parathyroidectomy" OR "Neck surgery" OR "endocrine surgery")) | 213 |
| **Cochrane** | Title-Abstract-Keywords (("Parathyroid Gland" OR "parathyroid") AND ("Thyroid Gland" OR "Thyroidectomy") AND ("Autofluorescence" OR "near infrared" OR "Near-Infrared" OR "NIR" OR "NIRAF" OR "fluorescence imaging" OR "Optical imaging" OR "Indocyanine Green" OR "ICG) AND ("Surgical Procedures" OR "Operative" OR "Surgery” OR "parathyroidectomy" OR "Neck surgery" OR "endocrine surgery")) | 68 |
| **Clinical trials.gov** | (("Parathyroid Gland" OR "parathyroid") AND ("Thyroid Gland" OR "Thyroidectomy") AND ("Autofluorescence" OR "near infrared" OR "Near-Infrared" OR "NIR" OR "NIRAF" OR "fluorescence imaging" OR "Optical imaging" OR "Indocyanine Green" OR "ICG) AND ("Surgical Procedures" OR "Operative" OR "Surgery” OR "parathyroidectomy" OR "Neck surgery" OR "endocrine surgery")) | 90 |
|  |  |  |

- **Date of Searching: January 2026**
- **Total: 562**
- **Duplicates: 171**
- **Total after removing duplicates: 391**

***Supplementary Table S2.*** *Quality assessment of the included observational studies utilizing the Newcastle-Ottawa Scale (NOS)*

(<https://www.ncbi.nlm.nih.gov/books/NBK115843/bin/appe-fm3.pdf>)

| **Studies** | **Selection** | | | | **Comparability** | | **Outcome** | | | **Score** | **AHRQ** |
| --- | --- | --- | --- | --- | --- | --- | --- | --- | --- | --- | --- |
|  | Representativeness of the exposed cohort | Selection of the non-exposed cohort | Ascertainment of exposure | Demonstration that outcome of interest was not present at start of study | Study controls of age | Study controls for additional important factor as gender | Assessment of outcome | Was follow-up long enough for outcomes to occur. | Adequacy of follow-up of cohorts. | **9** |  |
| **Barreira 2024** | * | * | * | * | *                 * | | * | * | * | 9 | Good |
| **Di Lorenzo 2024** | * | * | * | * | *                 * | | * | * | * | 9 | Good |
| **Hassan 2025** | * | * | * | * | *                 * | | * | * | * | 9 | Good |
| **Lahiri 2025** | * | * | * | * |  | | * | * | * | 7 | Poor |
| **Lau 2025** | * | * | * | * |  | | * | * |  | 6 | Poor |
| **Michaelsen 2025** | * | * | * | * | *                 * | | * | * | * | 9 | Good |
| **Tae 2025** | * | * | * | * | *                 * | | * | * | * | 9 | Good |

**Supplementary Figure S1.** *Risk of bias summary for the included randomized controlled trials evaluated by the Cochrane RoB 2 tool*

**Supplementary Figure S2.** *PRISMA Flow Diagram*

**Supplementary Table S3.** *GRADE evidence profile for primary and secondary outcomes*

| **Summary of findings** | | | | | | |
| --- | --- | --- | --- | --- | --- | --- |
| **Near-infrared (NIR) autofluorescence with indocyanine green (ICG) imaging compared to No near-infrared (NIR) autofluorescence without indocyanine green (ICG) imaging for patients who have undergone thyroid surgery or neck surgery** | | | | | | |
| **Patient or population:** Patients who have undergone thyroid surgery or neck surgery  **Setting:** Multicenter Hospitals  **Intervention:** near-infrared (NIR) autofluorescence with indocyanine green (ICG) imaging  **Comparison:** Non-near-infrared (NIR) autofluorescence without indocyanine green (ICG) imaging | | | | | | |
| Outcomes | **Anticipated absolute effects^*^** (95% CI) | | Relative effect (95% CI) | № of participants (studies) | Certainty of the evidence (GRADE) | Comments |
|  | **Risk with No near-infrared (NIR) autofluorescence without indocyanine green (ICG) imaging** | **Risk with near-infrared (NIR) autofluorescence with indocyanine green (ICG) imaging** |  |  |  |  |
| Transient hypoparathyroidism | 334 per 1,000 | **187 per 1,000** (70 to 511) | **RR 0.56** (0.21 to 1.53) | 908 (5 RCTs) | ⨁◯◯◯ Very low  ^a ,b^ | Downgraded 2 levels for inconsistency (I^2^=78%) and wide confidence intervals. |
| Permanent hypoparathyroidism | 174 per 1,000 | **78 per 1,000** (35 to 178) | **RR 0.45** (0.20 to 1.02) | 1325 (6 RCTs) | ⨁⨁⨁◯ Moderate  ^c^ | Downgraded for imprecision, because the confidence intervals are broad. |
| Transient hypocalcemia | 297 per 1,000 | **199 per 1,000** (98 to 398) | **RR 0.67** (0.33 to 1.34) | 792 (5 RCTs) | ⨁⨁◯◯ Low  ^d ,e^ | Downgraded for inconsistency (I^2^=75%) and wide confidence intervals. |
| Permanent hypocalcemia | 40 per 1,000 | **10 per 1,000** (7 to 15) | **RR 0.25** (0.17 to 0.37) | 350 (3 RCTs) | ⨁⨁◯◯ Low  ^f, g^ | Downgraded for Risk of Bias (unblinded outcome) and imprecision (OIS) not met |
| Inadvertent parathyroid removal | 149 per 1,000 | **64 per 1,000** (45 to 91) | **RR 0.43** (0.30 to 0.61) | 1917 (6 non-randomized studies) | ⨁⨁⨁⨁ High | Results were consistent (I^2^=0%) with precise estimates. |
| ***The risk in the intervention group** (and its 95% confidence interval) is based on the assumed risk in the comparison group and the **relative effect** of the intervention (and its 95% CI). **CI:** confidence interval; **RR:** risk ratio | | | | | | |
| **GRADE Working Group grades of evidence** **High certainty:** we are very confident that the true effect lies close to that of the estimate of the effect. **Moderate certainty:** we are moderately confident in the effect estimate: the true effect is likely to be close to the estimate of the effect, but there is a possibility that it is substantially different. **Low certainty:** our confidence in the effect estimate is limited: the true effect may be substantially different from the estimate of the effect. **Very low certainty:** we have very little confidence in the effect estimate: the true effect is likely to be substantially different from the estimate of effect. | | | | | | |

**Explanations**

a. Inconsistency: The study was downgraded since the heterogeneity (I^2) was high (78.4%) and the heterogeneity is statistically significant

b. Lack of precision: The 95% Confidence Interval is large and crosses null line of no impact (1.0) so that it includes both important benefit and important harm, which means that the data doesn't rule out either benefit or damage.

c. Lack of precision: The 95% Confidence Interval is large and crosses null line of no impact (1.0) so that it includes both important benefit and important harm, which means that the data doesn't rule out either benefit or damage.

d. Inconsistency: The study was downgraded since the heterogeneity (I^2) was substantial (75.4%) and the heterogeneity is statistically significant

e. Lack of precision: The 95% Confidence Interval is large and crosses null line of no impact (1.0) so that it includes both important benefit and important harm, which means that the data doesn't rule out either benefit or damage.

f. Risk of bias: The rating was downgraded because 50% of the studies included in the first and second domain raised "some concerns" This was primarily due to issues with the randomization process and, secondly regarding deviations from intended interventions or lack of blinding. When the operator is aware of the patient group undergoing near-infrared autofluorescence with indocyanine green, subjective outcomes are very likely to be biassed.

g. Lack of precision: Although the 95% Confidence Interval (CI) was narrow and did not cross the null line, the total number of patients and events recorded across the included studies was insufficient to meet the Optimal Information Size (OIS) requirements.
