# Supplementary material for: Indocyanine green (ICG) guided thyroidectomy versus conventional thyroidectomy and the impact on parathyroid function preservation: an updated GRADE-assessed systematic review and meta-analysis
Source: Langenbecks Arch Surg. 2026 Sep 1;411(1):257. doi: 10.1007/s00423-026-04210-3 (PMC13630843; doi:10.1007/s00423-026-04210-3)
Supplement: Supplementary file 4 [file 423_2026_4210_MOESM4_ESM.docx]

**Supplementary Table S4.** *Classification of included studies according to fluorescence imaging modality*

| **Study ID** | **Design** | **Fluorescence modality** | **Primary purpose of imaging** | **Notes** |
| --- | --- | --- | --- | --- |
| Yin et al., 2022 | RCT | Combined (NIRAF + ICG) | Identification (AF) + perfusion (ICG) | Dual-modality protocol |
| Rossi et al., 2023 | RCT | Combined (NIRAF + ICG) | Identification + perfusion | Both techniques used |
| Michaelsen et al., 2025 | Controlled trial | Combined (NIRAF + ICG) | Identification (NIRAF) + preresection perfusion (ICG) | Bimodal approach |
| Di Lorenzo et al., 2024 | Retrospective | NIRAF alone **and** Combined (NIRAF + ICG) | Identification (NIRAF) ± perfusion (ICG) | Three-arm study; fluorescence arms extracted as reported |
| Lau et al., 2025 | Retrospective cohort | NIRAF (autofluorescence) | Anatomical identification | ICG-capable endoscope used for autofluorescence (no dye injection for primary analysis) |
| Tae et al., 2025 | Retrospective matched | ICG angiography alone | Perfusion assessment | Transoral robotic thyroidectomy |
| Lahiri et al., 2025 | Ambispective | ICG fluorescence alone | Identification + perfusion scoring | Fluorescence intensity scored 1–3 |
| Hassan et al., 2025 | Retrospective matched | ICG-NIR (primarily ICG) | Identification + viability scoring | “Burjeel Protocol” |
| Barreira et al., 2024 | Retrospective | ICG angiography alone | Perfusion assessment & autotransplantation decision |  |
| Parfentiev et al., 2021 | RCT | ICG angiography | Identification + perfusion | Karl Storz NIR/ICG system |
| Grubnik et al., 2023 | RCT | ICG angiography | Identification + perfusion | Same group/system as Parfentiev |
