# Supplementary material for: Indocyanine green (ICG) guided thyroidectomy versus conventional thyroidectomy and the impact on parathyroid function preservation: an updated GRADE-assessed systematic review and meta-analysis
Source: Langenbecks Arch Surg. 2026 Sep 1;411(1):257. doi: 10.1007/s00423-026-04210-3 (PMC13630843; doi:10.1007/s00423-026-04210-3)
Supplement: Supplementary file 5 [file 423_2026_4210_MOESM5_ESM.docx]

**Supplementary Table S5. Outcome Definitions and Follow-up Criteria Across Included Studies**

| **Study ID** | **Transient Hypoparathyroidism / Hypocalcemia Definition** | **Permanent Hypoparathyroidism / Hypocalcemia Definition** | **Biochemical Thresholds** | **Follow-up Duration for Permanent Outcome** | **Notes** |
| --- | --- | --- | --- | --- | --- |
| Tae et al. (2025) | Transient hypoparathyroidism: postoperative serum iPTH <15 pg/mL on day of surgery or POD1.  Transient hypocalcemia: serum calcium <8.6 mg/dL on day of surgery or POD1. | Permanent: iPTH and calcium remaining below normal limits for >6 months. | iPTH lower limit 15 pg/mL; calcium lower limit 8.6 mg/dL (institutional). | >6 months | Measured pre-op, day of surgery, POD1, POD3, then 1, 3, 6, 12 months. |
| Yin et al. (2022) | Transient hypoparathyroidism: PTH <12 pg/mL postoperatively that recovered to normal within 6 months. | Permanent hypoparathyroidism: PTH remaining <12 pg/mL beyond 6 months. | PTH reference 12–88 pg/mL. Serum calcium also measured. | 6 months | Primary outcome was postoperative hypoparathyroidism (PTH <12 pg/mL on POD1). |
| Lahiri et al. (2025) | Hypocalcemia: ionized calcium <1.0 mmol/L on POD1. Hypoparathyroidism: PTH <7.5 pg/mL on POD1. | Not explicitly stratified as permanent in the reported outcomes (focus on POD1 rates). | Ionized Ca 1.0–1.3 mmol/L; PTH 7.5–53 pg/mL (institutional). | Not clearly defined for permanent (POD1 primary focus). | Ambispective design; historical controls. |
| Lau et al. (2025) | Postoperative hypocalcaemia: albumin-corrected calcium <2.1 mmol/L (secondary outcome in total thyroidectomy cases). | Not reported as a primary or secondary endpoint. | Albumin-corrected calcium <2.1 mmol/L. | Not applicable (no permanent outcome reported). | Focus on inadvertent parathyroidectomy; hypocalcaemia only for total thyroidectomy cases. |
| Michaelsen et al. (2025) | Not the primary focus (short-term AVDA use reported at discharge and 6 months). | Hypoparathyroidism at 1 year: treatment with an active vitamin D analogue (AVDA). | Defined by need for AVDA (not biochemical threshold alone). | 12 months | Primary outcome explicitly at 12 months using prescription registry cross-link. |
| Parfentiev et al. (2021) | Transient hypocalcemia: calcium <2.00 mmol/L observed on POD 5–10. | Permanent hypoparathyroidism/hypocalcemia: present at 3 months in 1 control patient. | Calcium <2.00 mmol/L for hypocalcemia. PTH also measured. | Assessed at 1, 3, and 6 months. | Calcium and PTH compared at POD1, POD 3–10, then 1, 3, 6 months. |
| Rossi et al. (2023) | Transient hypocalcemia: albumin-corrected calcium <8.0 mg/dL during hospitalization. Symptomatic hypocalcemia also defined. | Permanent (definitive) hypocalcemia: persistent at 6 months (most widely reported cutoff used). | Albumin-corrected calcium <8.0 mg/dL. | 6 months | All patients contacted and blood-tested at 6 months. |
| Di Lorenzo et al. (2024) | Transient hypoparathyroidism: PTH unmeasurable or <12 ng/mL (pg/mL) on POD1 (with or without hypocalcemia). | Permanent hypoparathyroidism: PTH below normal range persisting >6 months, requiring lifelong Ca + vitamin D. | PTH reference 12–65 pg/mL; corrected Ca 2.20–2.55 mmol/L. | >6 months | Standardized supplementation protocol based on POD1 PTH. |
| Grubnik et al. (2023) | Transient hypocalcemia: calcium <2.25 mmol/L on POD 5–10. | Permanent hypoparathyroidism/hypocalcemia: present at 3 months (2 patients in control group). | Calcium <2.25 mmol/L. | Assessed up to 6 months (permanent noted at 3 months). | Similar protocol to Parfentiev 2021 (same group). |
| Hassan et al. (2025) | Postoperative hypoparathyroidism stratified by PTH on POD1 (hypoparathyroidism vs euparathyroidism groups). | Not explicitly detailed beyond POD1 rates in the available text (focus on early outcomes and inadvertent parathyroidectomy). | Institutional PTH thresholds used for stratification on POD1. | Not fully detailed in extracted sections. | Matched retrospective cohort; emphasis on POD1 PTH and inadvertent parathyroidectomy. |
| Barreira et al. (2024) | Transient hypoparathyroidism reported (29.2% of patients with three score-2 glands still developed it). | Permanent hypoparathyroidism: 7% in non-ICG group vs 0% in ICG group. | Not fully specified in available abstract/text (ICG score used for perfusion). | Implied longer-term follow-up sufficient to classify permanent. | Focus on permanent hypoparathyroidism reduction with ICG angiography. |
