# Supplementary material for: Indocyanine green (ICG) guided thyroidectomy versus conventional thyroidectomy and the impact on parathyroid function preservation: an updated GRADE-assessed systematic review and meta-analysis
Source: Langenbecks Arch Surg. 2026 Sep 1;411(1):257. doi: 10.1007/s00423-026-04210-3 (PMC13630843; doi:10.1007/s00423-026-04210-3)
Supplement: Supplementary file 6 [file 423_2026_4210_MOESM2_ESM.docx]

**Figure 1. PRISMA flow diagram**
